# Small bowel diverticulum complicated by enterocutaneous fistula and abdominal wall abscess – Case report

**DOI:** 10.1016/j.ijscr.2019.02.037

**Published:** 2019-03-05

**Authors:** Mutlaq A. Almalki, Waed Y. Yaseen, Mohammed Baatiyyah

**Affiliations:** Department of General Surgery, Alnoor Specialized Hospital, Makkah, Saudi Arabia

**Keywords:** Small bowel diverticulum, Enterocutaneous fistula, Abdominal wall abscess

## Abstract

**Introduction:**

Small bowel diverticular disease is uncommon, representing 1–2% of general population. The ileum diverticulum is very rare, especially if it is complicated by enterocutaneous fistula with abdominal wall abscess.

**Presentation of the case:**

Here, we report a case of small bowel diverticulum, ileum diverticulum with enterocutaneous fistula in a 65-year man presented with lower abdominal pain and an irreducible swelling for 5 days.

**Dissection:**

Diverticulosis has no exact cause, but theoretically the diverticula result from peristalsis abnormalities, intestinal dyskinesia, or high segmental intraluminal pressures. Complications of small intestinal diverticulum include bleeding, diverticulitis and its complication (e.g. fistula formation, and intra-abdominal abscess) [5].

**Conclusion:**

This is considered rare and serious complication of small bowel diverticulum could occur if diagnosis is delayed.

## Highlights

• A small bowel diverticular disease is uncommon; the incidence is 1–2% of general population.
• Jejunal diverticula are more common and are larger than those in the ileum.
• One of very rare complication of small bowel diverticulum was ileal diverticulum with enterocutaneous fistula and abdominal wall abscess.
• The diverticulum was on the anti-mesenteric border instead of mesenteric border, which may explain the complication with enterocutaneous fistula.
• Enterolith can develop in the setting of intestinal stasis in the presence of intestinal diverticula.
• Bowel communication in abdominal wall abscess cases and use imaging modalities, like computed tomography, to optimize the pathway of the management.

## 1 Introduction

Diverticula are sac-like protrusions of the bowel wall and occur throughout the small and large bowel, usually from mesenteric border. A small bowel diverticular disease is uncommon; the incidence is 1–2% of general population [1]. The duodenal diverticula are more common than jejunoileal diverticula [2]. Jejunal diverticula are more common and are larger than those in the ileum, most of them are acquired diverticula occurring in patients at 6th and 7th decade of life. Majority of them are asymptomatic, may be diagnosed during diagnostic imaging for unrelated symptoms or intraoperative [3], however they may present with their signs and symptoms of complications. In our case, we reported one of very rare complication of small bowel diverticulum was ileal diverticulum with enterocutaneous fistula and abdominal wall abscess, associated with intestinal wall serosal lipoma and enterolith. We reported the case to discuss the etiopathogenesis and proper management of such cases. the work has been reported in line with the SCARE criteria. [4]

## 2 Case report

A 65-year-old Russian male, not known to have chronic medical illnesses, came to the ED complaining of painful swelling in the lower abdomen which had been going on for five days. Abdominal pain was severe colicky in nature with no relieving factors, associated with nausea and vomiting multiple times. There had been no change in bowel habits, fever or change in appetite. The patient had a history of lower abdominal surgery at the age of two, but he had no medical report

On physical examination the patient was conscious and had a normal body built. His blood pressure was 126/92, pulse was 88 and temperature was 36.2 °C. is symmetrically distended with a swelling in the lower abdomen 12 × 15 cm in size with negative cough impulse, erythema and tenderness on the overlying skin. The rest of the abdomen was soft on palpation with positive bowel sounds. Investigation of his hemoglobin gave 10.8 wbc’s with 11.5 sodium 139 potassium 3.2 creatinine 0.7.

**Fig. 1:**
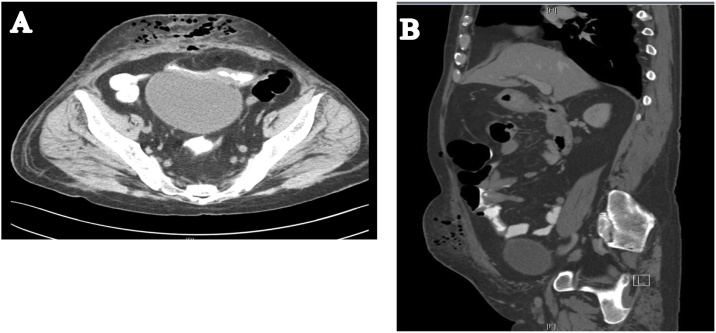
A and B, Abdomen CT with IV and oral contrast showed thicking of terminal ileum.

**Fig. 2:**
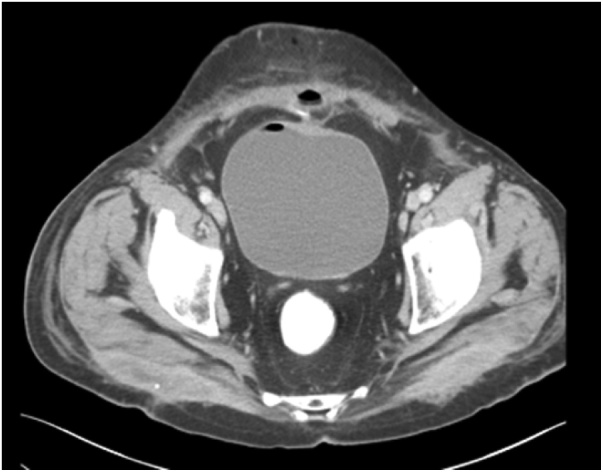
Abdominal CT with IV and oral contrast showed air pocket in bladder and ileo-cutaneous fistula.

The patient was admitted as a case of abdominal pain for investigation. The CT of abdomen and pelvic with IV and oral contrast was done showing thickened terminal ileum with marked luminal narrowing which appeared adherent to the urinary bladder wall with no line of cleavage. Two fistula tracts were seen superior and inferior; the superior one lead to a pocket of collection filled by contrast 36 × 20 mm in size. The inferior tract was connected to an anterior abdominal wall collection measuring about 18.7 × 14.4 mm with marginal enhancement denoting an abscess. There was diffuse anterior abdominal wall fat stranded with subcutaneous pockets of air denoting infection. Subcentemetric mesenteric lymphadenopathy was observed (Figs.1, 2).

**Fig. 3:**
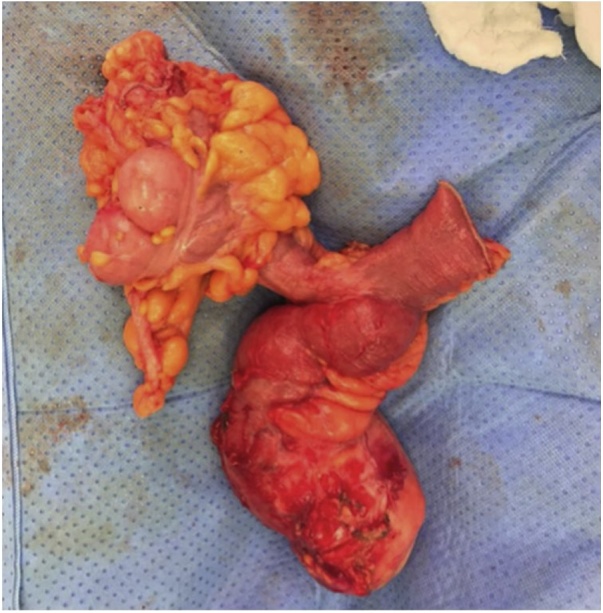
Resected bowel of 62 year old male patient, diagnosed with ileum diverticulum associated with abdominal wall abscess.

**Fig. 4:**
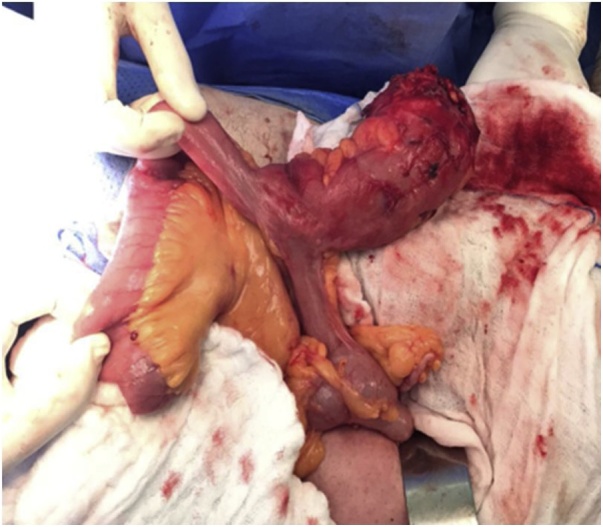
Terminal ileum diverticulum on antimesentric border.

Patient was taken to the OR for exploratory laparotomy and drainage of the abscess. Upon internce to the abdomen a large pocket of pus in subcutaneous layer was opened and evacuated and a swab was sent for culture and sensitivity. A firm mass inclosing the pelvic was dissected and found to be a large diverticulum 10 cm from the ileocecal junction. The mass was attaching to the urinary bladder and was fistulating to the subcutaneous pus collection. Urology was called in at this point and the urinary bladder was checked by injecting methylene blue dye; there was no leak. Limited right hemicolectomy was performed with a primary iliocolic anastomosis (Figs. 3,4).

**Fig. 5:**
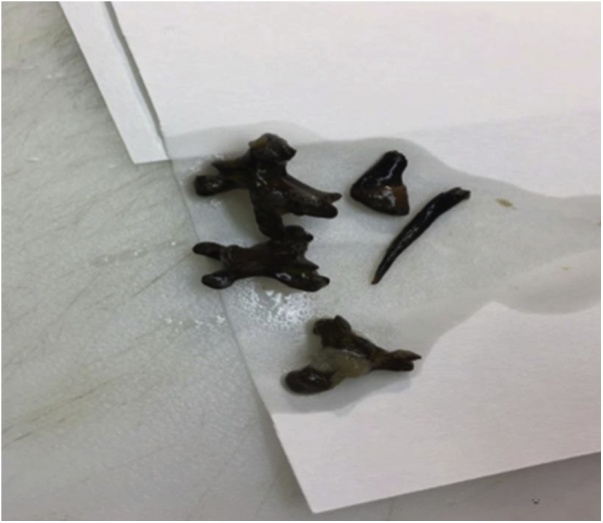
Enterolith looks as staghorn, found in the terminal ileum diverticulum.

Histopathology was consistent with diverticulum of the small bowel and serosal lipoma with a pocket containing multiple staghorn-type black stones, negative to tuberculosis (Fig. 5). Patient wound culture from OR showed E. coli which was sensitive to Tigacyclin. Treatment was started with this antibiotic and patient’s condition improved. Postoperative course was uneventful except for a small dehiscence at the lower part of the abdominal wound, which was treated conservatively with VAC dressing. Patient was discharged to travel to his country, and the wound was left for secondary closing.

## 3 Conclusion

We should consider the bowel communication in abdominal wall abscess cases and use imaging modalities, like computed tomography, to optimize the pathway of the management and decrease morbidity rate.

## 4 Discussion

Small intestinal diverticular disease is defined as out pouching of intestinal wall^6,^ usually from the mesenteric border^2^. It is uncommon; the incidence is 1–2% of general population^1^. Jejunoileal diverticula are less common than duodenal diverticula [2], occurring in 0.3%–1.3% of all cases of small bowel diverticular disease [1].

Diverticulosis has no exact cause, but theoretically the diverticula result from peristalsis abnormalities (e.g., intestinal spasms), intestinal dyskinesia, or high segmental intraluminal pressures. There are two types: A true type that is formed of all intestinal layer, and a false type, formed of just mucosa and submucosa [6]. Majority of them are asymptomatic, may be diagnosed during diagnostic imaging for unrelated symptoms or intraoperative [3] procedures; however, they may present signs and symptoms of complications. Complications of small intestinal diverticulum include bleeding, diverticulitis and its complication (e.g., obstruction, perforation, peritonitis, fistula formation, and intra-abdominal abscess), or segmental diverticulitis (e.g., inflammation in segments of the mucosal segments of colon in between diverticula) [6]. In the case reported here, the diverticulum was on the anti-mesenteric border instead of mesenteric border. In addition, there was the possibility of diverticula adhering to the abdominal wall due to the previous abdominal surgery, which can explain enterocutaneous fistula formation leading to an abdominal wall abscess rather than an intra-abdominal formed abscess.

In literature, abscess was drained percutaneous in Alvarez’s patient; however, resection and anastomosis of affected segment was the treatment for two Japanese patients (Eriguchi’s and Fujisawa’s) [6]. This was the treatment in present case and case of Meckel’s diverticulum reported by Oguzhan [3] which was also complicated by abdominal abscess. All of them had uneventful postoperative course.

An enterolith formation is not usually formed in a normal anatomy of bowel; however, it can develop in the setting of intestinal stasis in the presence of intestinal diverticula, surgical enteroanastomoses, blind pouches, afferent loops, incarcerated hernias, small intestinal tumors, intestinal kinking from intra-abdominal adhesions, and stenosis or stricture of Crohn’s disease and intestinal tuberculosis [7]. In our case there were two predisposing factors: history of enteroanastomosis and present ileal diverticulum. Therefore, it is suspected to be a primary result of intestinal stasis. Its prevalence ranges from 0.3% to 10% in selected populations [7]. Perforation is one of the rare complication of the enterolith. In our case we suppose the enterolith made compressions on the ileum diverticulum wall which was adherent to abdominal wall as a sequel of previous surgery or diverticulitis.

## Conflicts of interest

All authors of the research disclose that there are no financial and personal relationships with other people or organisations that could inappropriately influence (bias) their work.

## Funding

All authors of the research disclose that there are no sources of funding for our research.

## Ethical approval

The ethical approval for the publication of this case was exempted by our institution because all of the data were collected from clinical records and imaging systems for routine perioperative planning.

## Consent

Written informed consent was obtained from the patient for publication of this case report and accompanying images. A copy of the written consent is available for review by the Editor-in-Chief of this journal on request.

## Author contribution

MB and WA preformed the literature review, MB acquired and interpreted the data and drafted the manuscript. MA performed the operation and perioperative management of the patient. MB, WA participated in the operation, all authors participated perioperative management of the patient, postoperative-patient care, revision of the manuscript and gave final approval of the version of the manuscript to be published. All authors read and approved the final manuscript.

## Registration of research studies

Not relevant here.

## Guarantor

Mutlaq Almalki accept full responsibility for the work and had access to the data, and controlled the decision to publish.

## Provenance and peer review

Not commissioned, externally peer reviewed.
